# Maternal Personal Exposure to Airborne Benzene and Intrauterine Growth

**DOI:** 10.1289/ehp.0800465

**Published:** 2009-04-01

**Authors:** Rémy Slama, Olivier Thiebaugeorges, Valérie Goua, Lucette Aussel, Paolo Sacco, Aline Bohet, Anne Forhan, Béatrice Ducot, Isabella Annesi-Maesano, Joachim Heinrich, Guillaume Magnin, Michel Schweitzer, Monique Kaminski, Marie-Aline Charles, EDEN Mother–Child Cohort Study Group

**Affiliations:** 1 Inserm, Institut national de la santé et de la recherché médicale, University J Fourier Grenoble, Avenir Team “Environmental Epidemiology Applied to Fecundity and Reproduction,” U823, Institut Albert Bonniot, Grenoble, France; 2 University J. Fourier Grenoble, Grenoble, France; 3 Service de Gynécologie-Obstétrique, Maternité de Nancy, France; 4 Service de Gynécologie-Obstétrique, Centre Hospitalier Régional de Poitiers, France; 5 Institut national de la santé et de la recherche médicale, U822, “Epidémiologie, Démographie et Sciences Sociales,” Le Kremlin-Bicêtre, France; 6 University Paris-Sud, Le Kremlin-Bicêtre, France; 7 Fondazione Salvatore Maugeri–Centro di Ricerche Ambientali, Padova, Italy; 8 Institut national de la santé et de la recherche médicale, UMR 780, Villejuif, France; 9 Institut national de la santé et de la recherche médicale, EPAR, UMR-S707; 10 Faculté de Médecine de Saint-Antoine, UPMC Univ6, Paris, France; 11 Helmholtz Zentrum für Gesundheit und Umwelt, Helmholtz Zentrum München–German Research Center for Environmental Health, Institute of Epidemiology, Neuherberg, Germany; 12 Institut national de la santé et de la recherche médicale, UMRS 953, Epidemiological research on perinatal health and women’s and children’s health, Villejuif, France

**Keywords:** atmospheric pollution, benzene, birth weight, cohort, fetal growth, head circumference, personal monitoring, sensitivity analysis, ultrasonography

## Abstract

**Background:**

Studies relying on outdoor pollutants measures have reported associations between air pollutants and birth weight.

**Objective:**

Our aim was to assess the relation between maternal personal exposure to airborne benzene during pregnancy and fetal growth.

**Methods:**

We recruited pregnant women in two French maternity hospitals in 2005–2006 as part of the EDEN mother–child cohort. A subsample of 271 nonsmoking women carried a diffusive air sampler for a week during the 27th gestational week, allowing assessment of benzene exposure. We estimated head circumference of the offspring by ultrasound measurements during the second and third trimesters of pregnancy and at birth.

**Results:**

Median benzene exposure was 1.8 μg/m^3^ (5th, 95th percentiles, 0.5, 7.5 μg/m^3^). Log-transformed benzene exposure was associated with a gestational age–adjusted decrease of 68 g in mean birth weight [95% confidence interval (CI), −135 to −1 g] and of 1.9 mm in mean head circumference at birth (95% CI, −3.8 to 0.0 mm). It was associated with an adjusted decrease of 1.9 mm in head circumference assessed during the third trimester (95% CI, −4.0 to 0.3 mm) and of 1.5 mm in head circumference assessed at the end of the second trimester of pregnancy (95% CI, −3.1 to 0 mm).

**Conclusions:**

Our prospective study among pregnant women is one of the first to rely on personal monitoring of exposure; a limitation is that exposure was assessed during 1 week only. Maternal benzene exposure was associated with decreases in birth weight and head circumference during pregnancy and at birth. This association could be attributable to benzene and a mixture of associated traffic-related air pollutants.

Maternal exposure to air pollutants, and possibly traffic-related air pollutants during pregnancy, may influence fetal growth (Jedrychowski et al. 2004; Parker et al. 2005; Ritz and Wilhelm 2008; Slama et al. 2008a; Wilhelm and Ritz 2003). Traffic-related air pollution is a mixture of thousands of compounds present in gaseous form or as particulate matter (PM). These include aromatic hydrocarbons (e.g., benzene, polycyclic aromatic hydrocarbons), nonaromatic hydrocarbons (e.g., alkanes, olefins), metals, and inorganic gases such as nitrogen oxides and carbon monoxide (Krzyzanowski et al. 2005; Schauer et al. 1999, 2002). Most studies have focused on carbon monoxide (Ritz and Yu 1999), nitrogen dioxide, PM (Jedrychowski et al. 2004; Parker et al. 2005), and polycyclic aromatic hydrocarbons (Perera et al. 2005). With a few exceptions (e.g., Choi et al. 2006; Jedrychowski et al. 2004), exposure estimates were based on environmental models of outdoor air pollution levels close to the home address. These do not take into account the fact that outdoor levels of specific pollutants do not always reflect indoor levels, exposure in the workplace, and, importantly, levels in transit, which corresponds to a significant proportion of total personal exposure (Bruinen de Bruin et al. 2008; Ilgen et al. 2001b; Jo and Park 1999; Zhu et al. 2007). Therefore, studies relying on a personal exposure assessment are warranted.

For a few traffic-related air pollutants, animal experiments have reported effects of maternal exposure on fetal growth (Rocha et al. 2008). In rodents, airborne benzene exposure during pregnancy induces a reduction in fetal weight [Agency for Toxic Substances and Disease Registry (ATSDR) 2007]. In humans, studies of associations between benzene levels and pregnancy outcome have been conducted only in occupational settings (Chen et al. 2000; Wang et al. 2000), where benzene exposure is probably correlated with other chemicals than in the general population. Because of its antiknocking properties, benzene is used as an additive in gasoline; its presence in the atmosphere is attributable to industrial emissions and, predominantly, to motor vehicle traffic and combustion processes. Overall, the main sources of exposure in the general population are tobacco smoke, traffic, and other combustion processes (ATSDR 2007; Wallace 1996). For these reasons, benzene monitoring is a relevant candidate as a proxy measure of exposure to air pollutants related to traffic and to gasoline uses (Aguilera et al. 2008); moreover, it can be assessed by passive air samplers, which are light and relatively simple (Cocheo et al. 2000).

In studies on effects of air pollutants, fetal growth has most often been assessed by measures of birth weight, taking into account gestational duration (Glinianaia et al. 2004; Lacasana et al. 2005; Ritz and Wilhelm 2008; Slama et al. 2008a). One study reported a negative association between personal exposure to fine PM (aerodynamic diameter ≤2.5 μm; PM_2.5_) and head circumference (Jedrychowski et al. 2004), and another reported a possible effect of air pollution levels in early pregnancy on fetal ultrasound measurements, including head circumference (Hansen et al. 2008). Ultrasound measures constitute a promising approach to examine how early air pollution effects manifest in fetal growth (Hansen et al. 2008). Studying head size is particularly important, as head size is a marker of fetal growth that may specifically be associated with cognitive development in childhood (Yanney and Marlow 2004).

Our aim was to study, among nonsmoking women, the influence of personal exposure to benzene in the air, seen as a marker of traffic-related air pollution, on measures of fetal growth (birth weight, head circumference, biparietal diameter) assessed during pregnancy by ultrasonography and at birth.

## Population and Methods

### Study population

This study was conducted in a subgroup of the EDEN (study of pre- and early postnatal determinants of the child’s development and health) mother–child cohort (Drouillet et al. 2009). The primary aim of the EDEN cohort is the study of prenatal and early postnatal nutritional, environmental, and social determinants of children’s development and health. Women at < 20 gestational weeks (weeks of amenorrhea) were recruited from the maternity wards of Poitiers and Nancy University hospitals (France) between September 2003 and January 2006. Exclusion criteria were personal history of diabetes, multiple pregnancies, intention to deliver outside the university hospital or to move out of the study region within the next 3 years, and inability to speak French. We estimated participation rate among eligible women to be 55%. Supplemental Material, Table 1 (doi:10.1289/ehp.0800465.S1), compares our cohort with a national sample of women who delivered in 2003 (Blondel et al. 2006). Women were given an appointment with a study midwife, planned to take place between 24 and 28 gestational weeks, during which an interview on behavioral factors was conducted and biological samples were collected. For this study, we further restricted the EDEN cohort to nonsmoking women, who were asked by the study midwives to carry a diffusive air sampler for 7 consecutive days. For logistic reasons, this part of the study was offered only to women whose study visit took place after February 2005.

The study was approved by the relevant ethical committees (Comité Consultatif pour la Protection des Personnes dans la Recherche Biomédicale, Le Kremlin-Bicêtre University hospital, and Commission Nationale de l’Informatique et des Libertés), and all participating women gave informed written consent for themselves and for their child to be part of the study.

### Assessment of intrauterine growth

We assessed birth weight from maternity records. Head circumference was assessed at birth and also during a clinical examination of the newborn performed in duplicate by midwife research assistants within 3 days after birth for 95% of newborns. Our *a priori* choice was to use the average of these two postnatal measures; in sensitivity analyses, we also report the association between exposure and the single measure of head circumference at birth. We conducted ultrasound examinations between 29 and 36 gestational weeks (5th, 50th, and 95th percentiles, 30.7, 32.7, and 34.4 gestational weeks; third-trimester examination), between 19 and 27 gestational weeks (5th, 50th, and 95th percentiles, 20.7, 22.4, and 24.7 weeks; second-trimester examination), and before 15 gestational weeks (5th, 50th, and 95th percentiles, 11.2, 12.6, and 14.0 weeks; first-trimester examination). Because of the possible association between head circumference at birth and cognitive development in childhood (Yanney and Marlow 2004), and because of the availability of both pre- and postnatal measures of head circumference by different approaches, we *a priori* decided to focus on ultrasound measures of head circumference. In addition to the measurements at birth, we assessed head circumference during the second- and third-trimester (but not first-trimester) ultrasound examinations. Therefore, we also report associations of exposure with biparietal diameter (the widest diameter of the head), which we assessed at the first trimester examination; this was strongly correlated with head circumference during the second and third trimesters (coefficient of correlation, 0.92 during the second trimester and 0.80 during the third trimester, *p* < 10^−3^). Ultrasound measurements were performed according to Hadlock’s criteria (1994). Before the study start, the first five ultrasound measurements performed by each obstetrician were reviewed by one of us (O.T.). We assessed gestational duration at each examination and at birth from the date of the last menstrual period (LMP) (Slama et al. 2008b). When information on this date was missing or when the LMP-based gestational duration was > 44 gestational weeks, we used the obstetrician’s ultrasound-based estimate.

### Exposure to benzene

We used a diffusive air sampler (Radiello, Fondazione Salvatore Maugeri–Centro di Ricerche Ambientali, Padova, Italy) (Cocheo et al. 2000), which relies on radial symmetry diffusion (Cocheo et al. 1996). The cylindrical diffusive body contains a stainless steel net cylindrical cartridge, filled with activated charcoal. The absorbing cartridge was stored in a capped glass tube before and after the 7-day exposure period and sent by post to Institut national de la santé et de la recherche médicale (Inserm) by the participating woman after use, together with a questionnaire on the conditions of use. We excluded subjects for whom the diffusive part of the sampler was broken during use (*n* = 4). The charcoal cartridges were then temporarily stored and then shipped to the Maugeri Foundation, where they were stored at 4°C before analysis. Cartridges were shipped together with a bar code identifier and information on the hours and days of start and end of exposure to ambient air, but no information on pregnancy outcome. We desorbed the collected vapors from the cartridge using carbon disulfide solvent with a benzene concentration < 0.1 μg/mL and analyzed the solution using high-resolution gas chromatography with flame ionization detector. Taking into account the actual number of hours of exposure of the dosimeter, we converted the benzene concentration in the solution to a mean concentration in the air during the period of exposure. The detection limit for an exposure of 5 days is 0.1 μg/m^3^ benzene. Women were given illustrated instructions and were asked not to touch the diffusive air sampler with their hands, avoid contact with water, carry the air sampler always with them, attaching it on their clothes as close as possible to their collar, and to keep it close to their bed when they slept.

### Regression models

We studied the relationship between benzene and birth weight and the relationship between benzene and measures of head size assessed at birth and during the third trimester in distinct linear regression models (Stata SE version 10.1; StataCorp., College Station, TX, USA). We also studied the association between benzene exposure and measures of head size assessed during the first and second trimesters, assuming that benzene levels were indicative of exposure in early pregnancy. Benzene was considered either as a continuous variable, using the log-transformed values because of the skewed distribution of exposure, or as a variable whose categories corresponded to exposure tertiles defined on the whole population with an exposure estimate. We performed linear trend tests with a categorical variable whose values corresponded to the category-specific median benzene level.

Additionally, we conducted a longitudinal analysis including all three assessments of head circumference simultaneously, using multiple linear regression with a random effect variable corresponding to the mother–child pair and interaction terms with gestational age. We plotted the values of head circumference predicted by this longitudinal analysis as a function of benzene and gestational age, together with the observed values.

### Sensitivity analyses

We repeated the analyses among women who declared that their schedule during the week of use of the air sampler was similar to that during the previous month (usual schedule group). We also estimated the effect of benzene levels adjusting for the ultrasound-based gestational age instead of the LMP-based gestational age (Slama et al. 2008b). To determine whether an association between benzene and head circumference could be explained by an association between benzene and birth weight, we also estimated the effect of benzene on head circumference after excluding small-for-gestational-age births (10th percentile), using sex- and gestational age–specific references (Mamelle et al. 1996). Finally, we repeated analyses for various subgroups defined according to the conditions of use of the air sampler.

### Potential confounders

We selected confounders *a priori*, excluding all variables possibly affected by the health outcome or exposure (Rothman and Greenland 1998). We assessed exposure to passive smoking during the second trimester of pregnancy by a retrospective question filled in at birth asking whether someone smoked regularly in the presence of the woman, at home, in the workplace, or somewhere else. We also performed urinary cotinine measurements. Maternal urine was collected during the study visit at 24–28 gestational weeks and immediately frozen at −80°C. We then thawed a 1-mL aliquot and analyzed it by gas chromatography/mass spectrometry. We considered the group of women with a urinary cotinine level > 50 ng/mL (*n* = 5) to potentially include smokers and excluded them; additionally, we excluded two women who had declared to be nonsmokers during the interview with the midwife but who later declared to have smoked during the third trimester of pregnancy. Mean cotinine levels were 0.8 ng/mL among women who declared not to have been exposed to passive smoking and 2.3 ng/mL among women who declared to have been exposed to passive smoking (*p* < 0.01). In addition to passive smoking, cotinine levels, and gestational duration at the time of the measurement of fetal size (linear and quadratic terms), we also adjusted for sex of newborn, birth order, maternal height (continuous variable), prepregnancy weight [broken stick model with a knot at 60 kg (Slama and Werwatz 2005)], maternal age at end of study, and calendar month of conception. We also adjusted for maternal occupational exposure to paints, pesticides (*n* = 3, based on the questionnaire filled in between 24 and 28 gestational weeks), and center. We further adjusted models for head circumference at birth for cesarean sections (yes/no) and for the number of days between birth and the assessment of head circumference.

## Results

### Participants

A total of 2,002 pregnant women were recruited in the cohort. Among these women, 484 were nonsmoking women whose clinical examination took place after February 2005, and 304 women (63%) agreed to carry the dosimeter. Benzene levels and one measure of fetal growth were known for 271 women (89% of women who agreed to carry the dosimeter; overall participation rate within the cohort, 56%). Compared with approached nonsmoking women who refused to carry the air sampler or with no exposure estimate, those with an estimated benzene exposure were more often > 25 years of age and nulliparous at inclusion and more often declared to be exposed to passive smoking [Supplemental Material, Table 2 (doi:10.1289/ehp.0800465.S1)]. We found no evidence that participants and non-participants differed in terms of offspring birth weight or head circumference at any measurement (Student’s mean comparison test, all *p*-values > 0.29).

### Benzene levels

Participants carried the air sampler in the 27th week (median) of amenorrhea for a median duration of 7.0 days (Table 1). All benzene levels exceeded the detection limit, with a mean of 2.6 μg/m^3^ and a median of 1.8 μg/m^3^ (5th, 95th percentiles, 0.5, 7.5 μg/m^3^; range, 0.3–19.4 μg/m^3^); 26 (10%) and 6 values (2%) were > 5 and 10 μg/m^3^, respectively. Although we found no strong evidence of an association between exposure and the main means of transportation when exposure was categorized (Table 2), mean log-transformed exposure was higher for women using a car as the main means of transportation, compared with those walking, both before (*p* = 0.04) and after (*p* = 0.02) adjustment for month of measurement, center, and passive smoking. In unadjusted analyses, benzene levels tended to increase with gestational duration (*p* = 0.10).

### Benzene and birth weight

Median gestational week at delivery was 39.9 (5th, 95th percentiles, 35.6, 41.7 weeks). A benzene level in the highest exposure category was associated with an adjusted decrease in mean birth weight by 90 g [95% confidence interval (CI), −215 to 36 g], compared with the lowest exposure category. Each increase of one in log-transformed benzene exposure (corresponding to a multiplication of exposure by 2.72) was associated with an adjusted decrease of 68 g in birth weight (95% CI, −135 to −1 g; Table 3).

### Benzene and head circumference assessed at birth by the midwife

Compared with observations in the lowest exposure category, exposures in the intermediate and highest exposure categories were associated with adjusted decreases in mean head circumference at birth of 0.9 mm (95% CI, −4.5 to 2.7 mm) and 3.7 mm (95% CI, −7.3 to 0.0 mm), respectively (linear trend test, *p* = 0.04; Table 3). Log-transformed exposure showed a similar trend, corresponding to a decrease by 1.9 mm for each increase by one in log-transformed exposure (95% CI, −3.8 to 0.0; Table 3).

### Benzene and ultrasound measurements of head size

At the third-trimester ultrasonography, the adjusted decrease associated with benzene exposure in the highest exposure category was 4.8 mm for head circumference (95% CI, −8.8 to −0.8 mm, *p*-value for linear trend across exposure tertiles, 0.02) and 1.3 mm for biparietal diameter (95% CI, −2.6 to 0.1 mm; *p*-value for linear trend across tertiles, 0.02; Table 4). Log-transformed exposure showed consistent but statistically weaker associations with head circumference (*p* = 0.09) and biparietal diameter (*p* = 0.09).

At the second-trimester ultrasonography, the adjusted decrease in head circumference associated with exposure in the highest category was 2.5 mm (95% CI, −5.4 to 0.5 mm; *p*-value for linear trend across exposure categories, 0.11), and the adjusted decrease in biparietal diameter was 1.0 mm (95% CI, −2.0 to 0.0 mm; test for linear trend across categories, *p* = 0.06, Table 5). Each increase by one in log-transformed exposure was associated with an adjusted decrease by 1.5 mm in head circumference (95% CI, −3.1 to 0.0 mm, compared with −1.9 and −1.9 mm at birth and during the third trimester, respectively) and by 0.6 mm in biparietal diameter (95% CI, −1.1 to −0.1 mm) (Table 5).

Finally, at the first-trimester ultrasonography, compared with the lowest exposure category, the highest category was associated with an adjusted decrease in biparietal diameter by 0.9 mm (95% CI, −1.6 to −0.2 mm; *p*-value for linear trend, 0.03) (Table 6). Each increase by one in log-transformed exposure was associated with a decrease by 0.4 mm in biparietal diameter during the first trimester (95% CI, −0.7 to 0.0 mm, compared with −0.6 and −0.6 mm during the third and second trimesters, respectively).

### Longitudinal analysis

**Figure 1:**
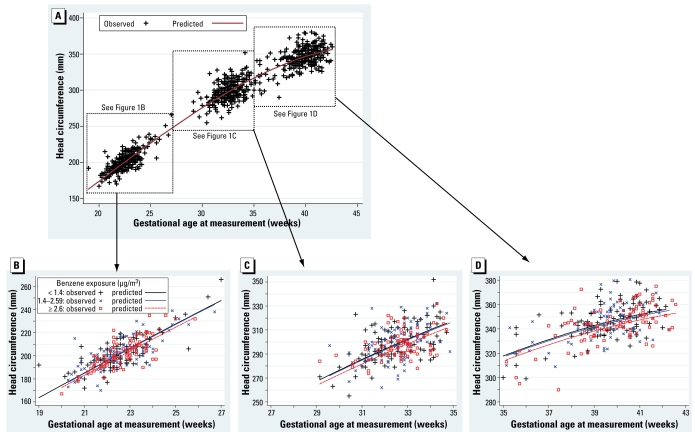
Head circumference as a function of gestational age at measurement and maternal benzene exposure. Head circumference was assessed between 19 gestational weeks and birth (*A*), between 19 and 27 gestational weeks (by ultrasonography) (*B*), between 27 and 35 gestational weeks (by ultrasonography) (*C*), and between 35 and 43 gestational weeks (after birth) (*D*). The predicted curves are adjusted for gestational age at examination (polynomial coding and interaction terms with all adjustment variables but education and center), sex, maternal passive smoking (questionnaire data), urinary cotinine level, prepregnancy weight, height, parity, maternal occupational exposure to paints or pesticides, month of conception, maternal education, and center.

Figure 1 shows the results of the longitudinal analysis combining second, third trimester, and birth measurements of head circumference, which are consistent with the above-described trimester-specific analyses.

### Sensitivity analyses

**Figure 2:**
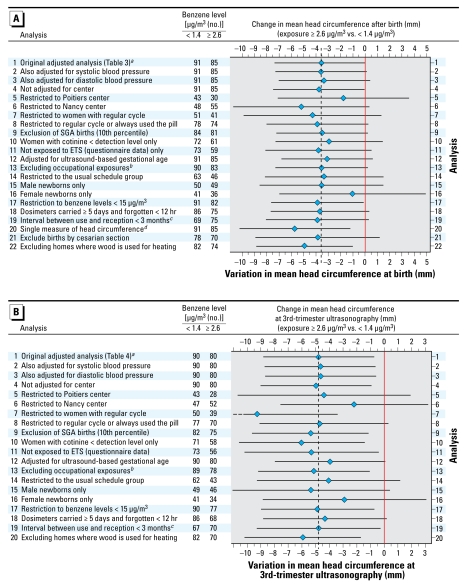
Sensitivity analysis: estimated effect of benzene levels (≥ 2.6 μg/m^3^, the reference being < 1.4 μg/m^3^) on head circumference after exclusion of specific subgroups of the population or adjustment for additional variables. Diamonds indicate point estimates; horizontal lines indicate the 95% CIs. Abbreviations: ETS, environmental tobacco smoke; SGA, small for gestational age. (*A*) Benzene and head circumference after birth (average of two measurements). (*B*) Benzene and head circumference at the third trimester (29–36 gestational weeks) ultrasound examination. *^a^*The sample sizes correspond to the adjusted analyses and thus slightly differ from those given in Table 3 or Table 4. *^b^*Exclusion of women occupationally exposed to paints or pesticides. *^c^*Exclusion of air samplers stored for ≥ 3 months during any period including a day in the warm season (June–September). *^d^*The single measure of head circumference performed right after birth was used instead of the average of the two independent measures performed shortly after birth.

When we restricted the analysis to the “usual schedule” group, the estimated birth weight change corresponded to a decrease of 87 g for each increase by one in log-transformed exposure (95% CI, −171 to −3 g; Table 3). Concerning head circumference and biparietal diameter, the results in the usual schedule group were similar to those in the whole population (Tables 3–6), although *p*-values were generally larger, with smaller sample sizes. The association with benzene was somewhat stronger when we assessed head circumference from the single measurement at birth (the variation in head circumference associated with the highest exposure category was −5.7 mm; 95% CI, −10.2 to −1.2 mm), compared with our original analysis using the average of two measurements within 3 days after birth (Figure 2A).

Associations remained similar after exclusion of subjects who lived in homes where wood or coal was used as a source of heating (a potential indoor source of benzene). We observed qualitatively similar results after exclusion of small-for-gestational-age births (Figure 2). Associations with head circumference were similar for subjects who had used the air samplers at least 5 days, for subjects who had not forgotten it in a room from which they were absent (or forgotten it for < 12 hr), and after exclusion of observations with an interval between the end of use of the sampler and storage in a refrigerator of > 90 days during part of the June–September period (Figure 2). As expected, associations tended to be weaker when we adjusted for ultrasound-based rather than LMP-based gestational duration (Figure 2). The estimated effect of benzene tended to be stronger among the group of 141 women who declared to have regular cycles, compared with results based on the whole population (Figure 2B).

## Discussion

In a cohort of nonsmoking women recruited during the first half of pregnancy, we observed decreased birth weight, decreased head circumference during the second and third trimesters of pregnancy and at birth, and decreased biparietal diameter during pregnancy in association with maternal benzene exposure.

Several studies in which exposure had been estimated from the air pollution levels in the vicinity of the home address had reported decrements in birth weight (corrected for gestational age) or increases in the risk of small-for-gestational-age births in association with air pollution levels (Jedrychowski et al. 2004; Parker et al. 2005; Ritz and Wilhelm 2008; Slama et al. 2008a; Wilhelm and Ritz 2003). Our study tends to confirm the association between air pollutants and gestational age–corrected birth weight using personal exposure assessment, adjusting for many potential confounders not always considered in previous studies, and focusing on a marker of air pollution specific of traffic and combustion sources.

Several studies have tried to identify windows of sensitivity to air pollutants during pregnancy, by testing associations between trimester-specific exposure variables and birth weight, without yielding a consistent picture (reviewed by Slama et al. 2008a). Only one study assessed fetal growth during pregnancy: In a study that assessed exposure from the air quality monitoring station data, Hansen et al. (2008) reported an association between the concentration of PM with an aerodynamic diameter < 10 μm and head circumference assessed by ultrasound between 13 and 26 gestational weeks. Their results and ours suggest that air pollutants could influence fetal head circumference during the second trimester of pregnancy. The fact that in our study benzene levels exhibited associations with biparietal diameter assessed at the end of the first trimester indicates that air pollution effects might manifest even earlier in pregnancy. However, this analysis should be considered with caution because the first- trimester examination was conducted at a time point more distant from benzene monitoring than the later ultrasound examinations.

Our analyses suggested a stronger association (measured on an additive scale) between benzene and fetal growth among male than among female newborns. Ghosh et al. (2007) suggested that air pollution effects on the risk of a low-birth-weight birth (assessed by multiplicative models) may differ by sex. In a cohort of pregnant women conducted in Poland, personal PM_2.5_ (PM with an aerodynamic diameter < 2.5 μm) exposure was more strongly associated with head circumference (using additive models) among male than among female newborns (Jedrychowski et al. 2009). Stronger effects of maternal smoking among male compared with female fetuses have also been reported, using either birth weight or biparietal diameter assessed by ultrasound as the outcome (Zaren et al. 2000). Our results are in line with these two studies (Jedrychowski et al. 2009; Zaren et al. 2000), relying on other measures of air pollution exposure.

Previous studies of associations between maternal benzene exposure and birth outcomes in human populations have been conducted in occupational settings, where benzene is used as a solvent and as an intermediate in the synthesis of many families of products (Chen et al. 2000; Wang et al. 2000). In a study in Taiwan, 792 women were recruited during pregnancy, among which 354 were considered occupationally exposed to benzene. After adjustment, a potential benzene exposure was associated with a nonsignificant reduction in mean birth weight of 15 g (95% CI, −52 to 82 g); in a model with an interaction term between benzene exposure and work stress, mean birth weight of offspring of the 57 women considered exposed to both factors was reduced by 183 g (95% CI, 65 to 301 g), compared with women considered exposed to neither factor (Chen et al. 2000). This study is difficult to compare with ours because it deals with occupational exposure, which generally stops during the third trimester of pregnancy (Chen et al. 2000), and because exposure assessment was performed on a binary scale by an industrial hygienist and did not consider nonoccupational exposures. Moreover, benzene exposure is probably a proxy for exposure to different mixtures of pollutants in occupational and nonoccupational settings.

The main strengths of our study are its prospective design, the use of ultrasound measurements to assess fetal growth, and the personal assessment of benzene exposure. The main limitation is that assessment was performed only once during 7 consecutive days, so we had no direct information on the variability in exposure during pregnancy. In addition, the sample size was too limited to study rare events such as occurrence of small-for-gestational-age births.

### Possible implications

Head circumference is correlated with brain volume in neonates (Lindley et al. 1999). The possible long-term consequences of air pollution effects on head circumference or brain volume are difficult to establish. A review concluded that children with poor prenatal head growth may be at risk for adverse neurodevelopmental outcomes (Yanney and Marlow 2004). Few studies have directly addressed the consequences of pre-natal exposure to air pollutants on cognitive development in childhood. Within a New York City, New York, cohort, prenatal exposure to polycyclic aromatic hydrocarbons was associated with lower mental development index at 3 years of age and a greater risk of cognitive developmental delay (Perera et al. 2006). However, this association remained after adjustment for birth weight and head circumference at birth (Perera et al. 2006), so any effect of polycyclic aromatic hydrocarbons on mental development is unlikely to be entirely mediated by changes in head circumference at birth.

### Benzene exposure as a proxy for exposure to traffic-related air pollutants

The mean personal benzene levels were 2.1 μg/m^3^ in the Poitiers area (a city of 100,000 inhabitants), compared with 2.9 μg/m^3^ in Nancy area, a more densely populated city of 300,000 inhabitants with higher mean environmental concentrations of NO_2_ estimated by the air quality monitoring stations (F. Caïni, Atmo Poitou-Charentes, personal communication). By comparison, mean exposure levels of 5.1 μg/m^3^ have been reported among non-smokers in Madrid, Spain, for the year 2003, and of 2.9 μg/m^3^ in Dublin, Ireland, in 2004 (Ballesta et al. 2006). Among our nonsmoking population, benzene exposure exceeded the European Union limit of 5 μg/m^3^ planned for the ambient air in 2010 for 26 subjects (10% of the population).

The main route of exposure to benzene is through the air, and the main sources of exposure among nonsmoking subjects from the general population are fuel and environmental tobacco smoke (Wallace 1996). Benzene is a recognized carcinogen, and emissions from most consumer products are expected to be very low. Although we cannot rule out the existence of indoor sources of benzene in the general population not related to fuel or cigarette smoke, the correlation between benzene exposure and outdoor NO_2_ levels does not speak in favor of important indoor sources of benzene. About 10% of women lived in a home where wood was used for heating; exclusion of these women did not modify the association between benzene and head circumference. Benzene is used as an additive in unleaded gasoline, in which a volume concentration of up to 1% is currently permitted in the United States and the European Union. Its presence in traffic exhaust is attributable to some benzene escaping the combustion process and benzene being a by-product of the partial combustion of other organic compounds. Benzene is also present in the environment because of other combustion processes such as residential heating or industrial emissions. Benzene levels much higher than common outdoor levels have been reported in car cabins (Ilgen et al. 2001b; Jo and Park 1999), so sitting in a car for about 1 hr/day can substantially increase personal exposure; a European study estimated that exposures in transit contribute to 29% of total personal benzene exposure (Bruinen de Bruin et al. 2008). Benzene environmental levels have been shown to be higher in the vicinity of roads with high traffic and in homes with a garage with a connecting door to the living rooms (Ilgen et al. 2001a). In our study, benzene exposure was higher for women who used the car as their main means of transportation. For these reasons, benzene exposure in our nonsmoking population in which effects of passive smoking have been controlled for can be seen as a proxy for exposure to traffic or combustion-related air pollutants.

### Possible biological mechanisms

There is support from experiments in rodents for an effect of maternal exposure to airborne benzene (not associated to other traffic-related air pollutants) on fetal weight [reviewed by ATSDR (2007)]. More probably, if it reflects a causal mechanism, the association that we observed could be attributable to a joint effect of several traffic-related air pollutants. In mice, effects on fetal weight have been reported for traffic exhaust exposure during pregnancy (Rocha et al. 2008; Veras et al. 2008). Several mechanisms could explain an effect of traffic-related air pollutants on fetal growth (Kannan et al. 2006; Slama et al. 2008a). These pollutants might influence endothelial function and blood viscosity, which could alter maternal–placental oxygen and nutrient exchanges and thus affect fetal growth (Slama et al. 2008a). This mechanism is supported by an experiment on mice that found effects of urban PM air pollution on placental morphology assessed, among other parameters, by the diameter of vessels (Veras et al. 2008). Endocrine disruption is another possible mechanism; indeed, endocrine disruption may play a role in the occurrence of intrauterine growth restriction and might be induced by diesel exhaust (Takeda et al. 2004).

### Assessment of fetal growth

The association between benzene exposure and head size at birth was stronger when we assessed head size from the single measurement right after birth than when we assessed it from the repeated measurements performed within a few days after birth, although trends were qualitatively similar. In additional analyses performed on the whole cohort, the estimated adjusted effect of maternal smoking was stronger for the single measure than for the average of the two measures (data not shown). The amplitude (but not the frequency) of measurement error is expected to be lower for the two measures than for the single measure. However, several factors that could not be adjusted for (e.g., postnatal nutrition of the newborn) might influence head size in the days after birth, so specific studies are warranted to determine which measure of head size is more relevant to characterize the influence of environmental factors on head circumference. In agreement with our expectation (Slama et al. 2008b), the estimated effect of exposure was stronger when we adjusted fetal size for gestational age based on the date of LMP, compared with based on the obstetrician estimate. The fact that the apparent effect of benzene tended to be stronger in the subgroup of women with a regular menstrual cycle than among the whole population, as highlighted by our sensitivity analyses, should be considered with caution because of the relatively small size of this subgroup. Although the direction of a bias due to measurement error on an adjustment factor is generally difficult to predict, this observation might be attributable to LMP-based gestational age being more efficiently assessed in the subgroup of women with regular cycles.

### Exposure assessment

Compared with approaches relying on measures in the environment, personal air samplers have the advantage of taking into account both indoor and outdoor exposures, as well as exposure in each microenvironment such as vehicle cabins. A limitation is that, unless repeated measurements are performed, they do not allow the capture of temporal variations in exposure. In our study, women carried the sampler at the same stage of fetal development and for a whole week to try to capture most of the usual situations of exposure. A German study with repeated measurements of indoor and outdoor benzene levels 6–13 months apart showed important intraindividual variation; however, after adjustment for season of measurement and region, the between-home standard deviation of benzene levels was greater than the within-home standard deviation, and the seasonal variations in benzene levels showed similar patterns across locations of measurement (Topp et al. 2004). Although the absolute value of exposure is expected to vary with time, it is plausible that the distribution of the population into exposure tertiles exhibits limited variations across time; that is, women in the highest exposure tertile at 27 gestational weeks may also correspond to a large proportion of the women in the highest tertile of exposure averaged over a longer period. In our study, restricting analyses to women with a time schedule during the week of use of the air sampler similar to that of the previous month was meant to focus on a subpopulation with more limited temporal variations in exposure due to behavioral factors, and hence an exposure estimate likely to be representative of exposure over a longer time period. This restriction yielded effect sizes of exposure similar to those estimated among the whole population.

We adjusted for both questionnaire-based exposure to passive smoking and cotinine urinary levels; our sensitivity analyses showed that excluding subjects exposed to passive smoking, as assessed by questionnaire or cotinine urinary levels, did not alter associations between benzene and head circumference, compared with adjusted analyses. However, we cannot firmly exclude any residual confounding due to passive smoking, because questionnaires have limited validity for low levels of exposure (Proctor et al. 1991), and because cotinine urinary assays may be limited by the relatively short half-life of cotinine among pregnant women. Half-life has been reported to be around 9 hr among pregnant women (Dempsey et al. 2002), compared with 17 hr after delivery (Dempsey et al. 2002) and 19 hr among male adults (Feng et al. 2007).

## Conclusion

We report an association between maternal pregnancy exposure of nonsmoking women to benzene and gestational age–adjusted offspring head size assessed at birth and by ultrasound imaging during pregnancy, and between exposure and birth weight. The association with head size manifested before the third trimester of pregnancy. Because benzene is a marker of exposure to traffic-related air pollutants, the association observed might be attributable to a mixture of traffic-related air pollutants, although we cannot completely rule out the existence of indoor sources of benzene not related to cigarette smoke or fuel.

## Figures and Tables

**Table 1:** Characteristics of use of the passive air sampler (271 nonsmoking women from the EDEN cohort).

		Percentile				
Characteristic	Mean	1st	5th	50th	95th	99th
Whole population (*n* = 271)						
Weeks of amenorrhea at the start of use	27.2	23	25	27	29	34
Duration of use (days)	7.2	3.0	6.8	7.0	8.2	14.1
Duration air sampler forgotten (hours)	4.3	0	0	0	9	36
Interval between end of use and reception by laboratory in charge of analysis (months)	2.3	0.3	0.4	2.1	5.2	6.8
Population with a usual schedule (*n* = 166)^a^						
Weeks of amenorrhea at the start of use	27.1	21	25	27	29	33
Duration of use (days)	7.2	1.6	6.9	7.0	8.0	14.1
Duration air sampler forgotten (hours)	4.6	0	0	0	36	36
Interval between end of use and reception by laboratory in charge of analysis (months)	2.2	0.3	0.4	2.0	4.7	5.2

a Restricted to women who declared that their schedule during the week of assessment of benzene exposure was similar to their schedule during the previous month.

**Table 2:** Characteristics of the participants and their association with benzene levels.

Characteristic	No. (%)	Mean (median) benzene level (μg/m^3^)	*p*-Value^a^	Benzene level [μg/m^3^ (%)]			*p*-Value^b^
				< 1.4 (*n* = 97)	1.4–2.59 (*n* = 87)	≥ 2.6 (*n* = 87)	
Sex of offspring			0.82				0.40

Male	147 (54)	2.6 (1.8)		37	29	34	
Female	124 (46)	2.6 (1.8)		34	36	30	

Gestational duration (weeks)			0.10^c^				0.02

27–36	23 (8)	2.3 (1.4)		48	30	22	
37–38	54 (20)	2.8 (1.9)		35	26	39	
39–40	143 (53)	2.5 (1.8)		36	39	25	
≥ 41	51 (19)	2.7 (2.3)		31	20	49	

Birth weight (g)			0.65				0.97

< 2,500	21 (7)	3.0 (2.2)		33	33	33	
≥ 2,500	249 (91)	2.5 (1.8)		36	32	32	
Missing value	1 (0.3)	2.7 (2.7)					

Birth order			0.43^c^				0.53

First birth	129 (48)	2.6 (1.8)		38	33	29	
Second birth	89 (33)	2.7 (1.8)		38	28	34	
Third birth or more	53 (20)	2.3 (2.0)		26	37	36	

Maternal age at conception (years)			0.88				0.97

< 25	39 (14)	2.5 (1.7)		36	28	36	
25–29	108 (40)	2.9 (1.8)		35	32	32	
30–34	81 (30)	2.6 (1.9)		35	32	33	
≥ 35	43 (16)	2.3 (1.8)		40	35	26	

Maternal height (cm)			0.71				0.88

< 160	75 (27)	2.3 (1.8)		35	35	31	
160–169	152 (56)	2.7 (1.8)		34	32	34	
≥ 170	41 (15)	2.7 (1.8)		41	29	29	
Missing value	3 (1)	0.6 (0.3)					

Maternal prepregnancy weight (kg)			0.26				0.38

< 50	27 (10)	2.7 (1.9)		41	26	33	
50–59	114 (42)	2.2 (1.6)		42	31	27	
60–69	81 (30)	2.9 (2.1)		23	38	38	
70–79	30 (11)	3.3 (1.9)		40	27	33	
≥ 80	19 (7)	2.1 (1.6)		37	32	32	

Maternal age at end of education (years)			0.15^c^				0.06

≤ 17	14 (5)	2.7 (2.4)		14	36	50	
18–19	45 (17)	2.6 (2.0)		33	29	37	
20–21	53 (19)	2.7 (1.8)		25	43	32	
22–23	80 (29)	2.4 (1.5)		49	23	29	
> 23	79 (29)	2.6 (1.8)		35	35	29	

Urinary cotinine level (ng/mL)			0.11^c^				0.57

< Limit of detection	209 (77)	2.5 (1.7)		37	33	30	
0.1–5	48 (18)	2.5 (1.9)		33	31	35	
> 5	14 (5)	3.0 (2.5)		21	29	50	

Maternal passive smoking (second trimester)			0.07				0.25

No	211 (78)	2.5 (1.8)		38	31	31	
Yes	60 (22)	2.9 (2.1)		27	37	37	

Month of benzene measurement			< 10^−4^				< 10^−3^

January–March	91 (34)	2.8 (2.3)		21	35	44	
April–June	68 (25)	2.1 (1.5)		47	26	26	
July–September	65 (24)	2.5 (1.0)		65	18	17	
October–December	47 (17)	2.8 (2.2)		9	53	38	

Month of conception of the child			< 10^−4^				< 10^−3^

January–March	66 (24)	2.0 (1.0)		65	23	12	
April–June	42 (15)	3.2 (2.2)		12	52	36	
July–September	80 (30)	2.9 (2.3)		15	38	48	
October–December	83 (30)	2.4 (1.6)		45	24	31	

Use of wood or coal for heating			0.82				0.30

No	237 (87)	2.6 (1.8)		36	33	31	
Yes	25 (9)	2.3 (2.2)		36	20	44	
Missing value	9 (3)	3.1 (2.4)					

Most frequent means of transportation^d^			0.06				0.29

Car	199 (73)	2.7 (1.9)		32	34	34	
Bus	10 (4)	2.6 (2.2)		20	40	40	
Walk	47 (17)	2.1 (1.3)		51	26	23	
Other	14 (5)	2.1 (1.5)		43	29	29	
Missing value	1 (0.4)	0.3 (0.3)					

Daily time spent in a car (min)^e^			0.71				0.72

< 30	142 (52)	2.6 (1.8)		35	33	32	
30–59	77 (28)	2.2 (1.7)		40	27	32	
≥ 60	47 (17)	3.2 (2.0)		30	38	32	
Missing value	5 (2)	1.7 (2.0)					

NO_2_ estimate at home address (μg/m^3^ ) f			< 10^−3^				< 10^−3^

< 18.44	89 (33)	2.1 (1.4)		55	19	26	
18.45–29.39	88 (32)	2.1 (1.8)		35	35	30	
> 29.40	88 (32)	2.8 (2.2)		18	43	39	
Missing value	6 (2)	2.6 (2.8)					

PM_10_ estimate at home address (μg/m^3^ )^f^			0.02				0.02

< 16.37	84 (31)	2.6 (1.7)		42	27	31	
16.37–20.69	86 (32)	2.2 (1.5)		45	34	21	
> 20.70	84 (31)	2.9 (2.2)		24	36	40	
Missing value	17 (6)	2.8 (2.6)					

Home with windows opening on the street			0.003				0.02

No	58 (21)	1.7 (1.4)		52	26	22	
Yes	207 (76)	2.8 (1.9)		32	33	35	
Missing value	6 (2)	2.1 (2.3)					

Center			0.005				0.05

Nancy	160 (59)	2.9 (2.0)		30	36	34	
Poitiers	111 (41)	2.1 (1.6)		44	27	29	

a *p*-Value of Kruskal–Wallis rank test (or trend test if specified). The test was performed excluding the category corresponding to missing values.

b *p*-Value of chi-square test (exact test when required). The test was performed excluding the category corresponding to missing values.

c *p*-Value of nonparametric trend test by Cuzick (1985).

d During the week before the start of use of the passive air sampler.

e The value was set to 0 for women for whom car was not the most frequent means of transportation.

f Average during the period of use of the passive air sampler of the measurements from the permanent monitoring station closest from the home address.

**Table 3:** Associations between benzene levels during pregnancy and measurements of the offspring at birth.

			Gestational age–adjusted models^a^		Fully adjusted models^b^	
Measure	No.	Mean ± SD	β^c^ (95% CI)	*p*-Value	β^c^ (95% CI)	*p*-Value
Birth weight (g)						
Benzene exposure, whole population (μg/m^3^ )						
< 1.4	97	3,309 ± 574	0	0.59^d^	0	0.23^d^
1.4–2.59	87	3,262 ± 555	−77 (−195 to 41)	0.20	−74 (−197 to 50)	0.24
≥ 2.6	86	3,336 ± 497	−45 (−163 to 74)	0.46	−90 (−215 to 36)	0.16
ln(benzene)	270	3,302 ± 543	−40 (−102 to 21)	0.20	−68 (−135 to −1)	0.05
Benzene exposure, population with a usual schedule (μg/m^3^ )^e^						
< 1.4	65	3,354 ± 549	0	0.34^d^	0	0.19^d^
1.4–2.59	53	3,386 ± 560	−7 (−157 to 142)	0.92	13 (−150 to 177)	0.87
≥ 2.6	46	3,375 ± 470	−73 (−230 to 84)	0.36	−95 (−257 to 68)	0.25
ln(benzene)	164	3,370 ± 529	−59 (−141 to 23)	0.16	−87 (−171 to −3)	0.04

Head circumference after birth (mm)						
Benzene exposure, whole population (μg/m^3^ )						
< 1.4	94	345.8 ± 16	0	0.10^d^	0	0.04^d^
1.4–2.59	86	344.6 ± 15	−1.3 (−5.0 to 2.3)	0.47	−0.9 (−4.5 to 2.7)	0.32
≥ 2.6	85	344.0 ± 16	−3.1 (−6.7 to 0.5)	0.10	−3.7 (−7.3 to 0.0)	0.05
ln(benzene)	265	344.8 ± 16	−1.4 (−3.3 to 0.4)	0.14	−1.9 (−3.8 to 0.0)	0.06
Benzene exposure, population with a usual schedule (μg/m^3^ )^e^						
< 1.4	64	346.6 ± 13	0	0.06^d^	0	0.08^d^
1.4–2.59	52	347.3 ± 16	0.5 (−3.9 to 4.9)	0.82	2.1 (−2.8 to 6.9)	0.40
≥ 2.6	46	344.1 ± 13	−4.2 (−8.8 to 0.4)	0.07	−3.3 (−8.1 to 1.5)	0.17
ln(benzene)	162	346.1 ± 14	−1.9 (−4.3 to 0.5)	0.12	−2.2 (−4.7 to 0.4)	0.09

Model’s adjusted *R*^2^ (whole population only, models with log-transformed exposure) was 0.54 for birth weight and also 0.54 for head circumference after birth.

a Adjusted for gestational age at birth (linear and quadratic terms).

b Adjusted for gestational age at birth (linear and quadratic terms), sex, maternal passive smoking (questionnaire data), urinary cotinine levels (three categories), prepregnancy weight, height, birth order, occupational exposure to paints or pesticides, month of conception, maternal age at end of studies, and center. Models for head circumference were further adjusted for cesarian birth (yes/no), and the number of days between birth and measurement of head size.

c Parameter of the linear regression model associated with benzene, corresponding to the difference in mean birth weight (g) or head circumference (mm) with respect to the first exposure category; for the continuous coding, β corresponds to the change in mean birth weight (g) or head circumference (mm) for each increase by one in log-transformed exposure.

d *p*-Value for linear trend across exposure categories.

e Restricted to women who declared that their schedule during the week of assessment of benzene exposure was similar to their schedule from the previous month.

**Table 4:** Association between benzene levels during pregnancy and head ultrasound measurements during the third trimester of pregnancy.

			Gestational age–adjusted models^a^		Fully adjusted models^b^	
Measure	No.	Mean ± SD (mm)	β^c^ (95% CI)	*p*-Value	β^c^ (95% CI)	*p*-Value
Head circumference at the third-trimester ultrasound examination						
Benzene exposure, whole population (μg/m^3^ )						
< 1.4	93	298.7 ± 15	0	0.15^d^	0	0.02^d^
1.4–2.59	86	299.0 ± 15	−0.9 (−4.6 to 2.7)	0.61	−1.6 (−5.4 to 2.3)	0.43
≥ 2.6	80	297.6 ± 11	−2.7 (−6.3 to 1.0)	0.16	−4.8 (−8.8 to −0.8)	0.02
ln(benzene)	259	298.5 ± 14	−0.5 (−2.5 to 1.4)	0.58	−1.9 (−4.0 to 0.3)	0.09
Benzene exposure, population with a usual schedule (μg/m^3^ )^e^						
< 1.4	63	298.9 ± 16	0	0.09^d^	0	0.06^d^
1.4–2.59	52	300.5 ± 14	1.1 (−3.4 to 5.5)	0.63	2.6 (−2.7 to 7.8)	0.33
≥ 2.6	43	296.5 ± 10	−3.8 (−8.5 to 0.9)	0.11	−4.1 (−9.3 to 1.2)	0.13
ln(benzene)	158	298.8 ± 14	−0.5 (−3.0 to 2.0)	0.69	−1.8 (−4.6 to 1.0)	0.21

Biparietal diameter at the third-trimester ultrasound examination						
Benzene exposure, whole population (μg/m^3^ )						
< 1.4	93	83.2 ± 4.8	0	0.07^d^	0	0.02^d^
1.4–2.59	86	83.3 ± 4.2	−0.2 (−1.3 to 0.9)	0.66	−0.2 (−1.5 to 1.0)	0.69
≥ 2.6	81	82.8 ± 3.7	−1.0 (−2.1 to 0.1)	0.08	−1.3 (−2.6 to −0.1)	0.04
ln(benzene)	260	83.1 ± 4.3	−0.3 (−0.9 to 0.3)	0.27	−0.6 (−1.2 to 0.1)	0.09
Benzene exposure, population with a usual schedule (μg/m^3^ )^e^						
< 1.4	63	83.4 ± 5.3	0	0.12^d^	0	0.09^d^
1.4–2.59	52	83.8 ± 4.2	0.2 (−1.2 to 1.7)	0.76	0.5 (−1.2 to 2.2)	0.59
≥ 2.6	44	82.9 ± 4.0	−1.1 (−2.6 to 0.4)	0.15	−1.2 (−2.9 to 0.5)	0.15
ln(benzene)	159	83.4 ± 4.6	−0.3 (−1.1 to 0.5)	0.43	−0.7 (−1.6 to 0.2)	0.14

Model’s adjusted *R*^2^ (whole population only, models with log-transformed exposure) was 0.32 for head circumference and 0.35 for biparietal diameter.

a Adjusted for gestational age at examination (linear and quadratic terms).

b Adjusted for gestational age at the examination (linear and quadratic terms), sex, maternal passive smoking (questionnaire data), urinary cotinine levels (three categories), prepregnancy weight, height, parity, occupational exposure to paints or pesticides, month of conception, maternal education, and center.

c Parameter of the linear regression model associated with benzene, corresponding to the difference in mean head size expressed in millimeters with respect to the first exposure category or, for the continuous coding, to the change in mean head size for each increase by one in log-transformed exposure.

d *p*-Value for linear trend across exposure categories.

e Restricted to women who declared that their schedule during the week of assessment of benzene exposure was similar to their schedule from the previous month.

**Table 5:** Association between benzene levels during pregnancy and head ultrasound measurements during the second trimester of pregnancy.

			Gestational age–adjusted models^a^		Fully adjusted models^b^	
	No.	Mean ± SD (mm)	β^c^ (95% CI)	*p*-Value	β^c^ (95% CI)	*p*-Value
Head circumference at the second-trimester ultrasound examination						
Benzene exposure, whole population (μg/m^3^ )						
< 1.4	95	202.5 ± 14	0	0.45^d^	0	0.11^d^
1.4–2.59	83	201.8 ± 14	−0.7 (−3.4 to 1.9)	0.59	−1.3 (−4.2 to 1.6)	0.37
≥ 2.6	81	202.2 ± 13	−1.1 (−3.8 to 1.6)	0.43	−2.5 (−5.4 to 0.5)	0.10
ln(benzene)	259	202.2 ± 14	−0.6 (−2.0 to 0.8)	0.41	−1.5 (−3.1 to 0.0)	0.05
Benzene exposure, population with a usual schedule (μg/m^3^ )^e^						
< 1.4	60	203.7 ± 16	0	0.71^d^	0	0.20^d^
1.4–2.59	52	201.7 ± 13	1.0 (−2.5 to 4.4)	0.58	1.0 (−3.1 to 5.2)	0.62
≥ 2.6	44	202.6 ± 14	−0.5 (−4.2 to 3.1)	0.77	−2.2 (−6.2 to 1.8)	0.28
ln(benzene)	156	202.8 ± 14	−0.3 (−2.2 to 1.6)	0.76	−1.6 (−3.8 to 0.5)	0.14

Biparietal diameter at the second-trimester ultrasound examination						
Benzene exposure, whole population (μg/m^3^ )						
< 1.4	95	55.3 ± 4.5	0	0.65^d^	0	0.06^d^
1.4–2.59	86	55.3 ± 4.5	−0.2 (−1.1 to 0.7)	0.73	−0.5 (−1.5 to 0.5)	0.32
≥ 2.6	84	54.9 ± 4.0	−0.5 (−1.4 to 0.4)	0.29	−1.0 (−2.0 to 0.0)	0.05
ln(benzene)	265	55.2 ± 4.3	−0.3 (−0.7 to 0.2)	0.27	−0.6 (−1.1 to −0.1)	0.02
Benzene exposure, population with a usual schedule (μg/m^3^ )^e^						
< 1.4	63	55.5 ± 4.9	0	0.65^d^	0	0.21^d^
1.4–2.59	53	55.1 ± 4.0	0.1 (−1.0 to 1.3)	0.81	−0.1 (−1.5 to 1.3)	0.91
≥ 2.6	44	55.0 ± 4.4	−0.3 (−1.5 to 1.0)	0.69	−0.8 (−2.2 to 0.6)	0.24
ln(benzene)	160	55.2 ± 4.4	−0.2 (−0.8 to 0.5)	0.62	−0.6 (−1.4 to 0.1)	0.09

Model’s adjusted *R*^2^ (whole population only, models with log-transformed exposure) was 0.64 for head circumference and 0.56 for biparietal diameter.

a Adjusted for gestational age at examination (linear and quadratic terms).

b Adjusted for gestational age at the examination (linear and quadratic terms), sex, maternal passive smoking (questionnaire data), urinary cotinine levels (three categories), prepregnancy weight, height, parity, maternal occupational exposure to paints or pesticides, month of conception, maternal education, and center.

c Parameter of the linear regression model associated with benzene, corresponding to the difference in mean head size expressed in millimeters with respect to the reference category or, for the continuous coding, to the change in mean head size for each increase by one in log-transformed exposure.

d *p*-Value for linear trend across categories.

e Restricted to women who declared that their schedule during the week of assessment of benzene exposure was similar to their schedule from the previous month

**Table 6:** Association between benzene levels during pregnancy and ultrasound measurements of biparietal diameter during the first trimester of pregnancy.

			Gestational age–adjusted models^a^		Fully adjusted models^b^	
Benzene exposure	No.	Mean ± SD (mm)	β^c^ (95% CI)	*p*-Value	β^c^ (95% CI)	*p*-Value
Whole population (μg/m^3^ )						
< 1.4	84	22.1 ± 2.9	0	0.11^d^	0	0.03^d^
1.4–2.59	85	21.6 ± 3.2	−0.5 (−1.2 to 0.1)	0.10	−0.7 (−1.4 to 0.0)	0.05
≥ 2.6	84	21.9 ± 3.2	−0.6 (−1.2 to 0.1)	0.08	−0.9 (−1.6 to −0.2)	0.01
ln(benzene)	253	21.9 ± 3.1	−0.2 (−0.5 to 0.2)	0.30	−0.4 (−0.7 to 0.0)	0.06

Population with a usual schedule (μg/m^3^ )^e^						
< 1.4	57	21.9 ± 2.8	0	0.42^d^	0	0.25^d^
1.4–2.59	50	21.9 ± 3.1	−0.1 (−0.9 to 0.7)	0.83	−0.3 (−1.2 to 0.7)	0.58
≥ 2.6	44	22.5 ± 3.4	−0.4 (−1.2 to 0.5)	0.42	−0.6 (−1.5 to 0.4)	0.24
ln(benzene)	151	22.1 ± 3.1	0.0 (−0.5 to 0.4)	0.85	−0.2 (−0.7 to 0.3)	0.37

Model’s adjusted *R*^2^ (whole population only, model with log-transformed exposure) was 0.57.

a Adjusted for gestational age at examination (linear and quadratic terms).

b Adjusted for gestational age at the examination (linear and quadratic terms), sex, maternal passive smoking (questionnaire data), urinary cotinine levels (three categories), prepregnancy weight, height, parity, maternal occupational exposure to paints or pesticides, month of conception, maternal education, and center.

c Parameter of the linear regression model associated with benzene, corresponding to the difference in mean biparietal diameter expressed in millimeters with respect to the reference category or, for the continuous coding, to the change in mean biparietal diameter for each increase by one in log-transformed exposure.

d *p*-Value for linear trend across exposure categories.

e Restricted to women who declared that their schedule during the week of assessment of benzene exposure was similar to their schedule from the previous month.
